# Efficacy of platelet rich fibrin in the reduction of the pain and swelling after impacted third molar surgery: Randomized multicenter split-mouth clinical trial

**DOI:** 10.1186/s13005-015-0094-5

**Published:** 2015-11-26

**Authors:** Ozkan Ozgul, Fatma Senses, Nilay Er, Umut Tekin, Hakan Hıfzı Tuz, Alper Alkan, Ismail Doruk Kocyigit, Fethi Atil

**Affiliations:** Department of Oral and Maxillofacial Surgery, Faculty of Medicine, Ufuk University, Ankara, Turkey; Department of Oral and Maxillofacial Surgery, Faculty of Dentistry, Kırıkkale University, Kırıkkale, Turkey; Department of Oral and Maxillofacial Surgery, Faculty of Dentistry, Trakya University, Edirne, Turkey; Department of Oral and Maxillofacial Surgery, Faculty of Dentistry, Hacettepe University, Ankara, Turkey; Department of Oral and Maxillofacial Surgery, Faculty of Dentistry, Erciyes University, Kayseri,, Turkey

**Keywords:** PRF, Third molar surgery, Pain, Swelling

## Abstract

**Background:**

Impacted third molar removal is a routine procedure in oral and maxillofacial surgery. Platelet-rich fibrin (PRF) is a second generation platelet concentration which is produced by simplified protocol. The aim of this study was to assess the effectiveness of PRF in the healing process by evaluating the changes in pain and swelling after third molar surgery.

**Methods:**

Fifty-six patients (23 male, 33 female) who provide the inclusion criteria were selected to participate in this study. The evaluation of the facial swelling was performed by using a horizontal and vertical guide. The pain was evaluated in the postoperative period using a visual analog scale (VAS) of 100 mm.

**Results:**

Horizontal and vertical measurements showed more swelling at the control side (without PRF) in 3th day postoperatively (*p* < 0.05). There were no statistically significant differences regarding pain among the groups.

**Conclusion:**

As a conclusion, PRF seems to be effectiveness on postoperative horizontal swelling after third molar surgery. PRF could be used on a routine basis after third molar extraction surgery.

## Background

The removal of impacted third molars is common oral surgical procedure. Surgical extraction of third molars is often accompanied by pain, swelling, trismus, and general oral dysfunction during the healing period [1–5]. Careful surgical technique and scrupulous perioperative care can minimize the frequency of complications and limit their severity [2].

Various pharmacological and/or extraction methods have been used for maintaining patients social activities. These include non-steroid anti-inflammatory drugs (NSAIDs), laser treatment, steroids and ultrasound [6–9]. However, the amount and intensity of edema, pain and trismus occurring after surgical extraction cannot be eliminated completely.

Platelet-rich fibrin (PRF) is a second generation platelet concentration which is produced by simplified protocol. PRF consists of a fibrin matrix polymerized in a tetramolecular structure, the incorporation of platelets, leukocyte, and cytokines, and the presence of circulating stem cells [10–12]. There are many studies showing accelerating wound healing of PRF in periodontal defects, cyst cavities and sinus floor augmentation in the literature [13–15]. There are limited studies on the effects of PRF on postoperative pain and swelling [16, 17].

The aim of this study is to evaluate the incidence and severity of postoperative swelling and pain following mandibular third molar surgery using PRF as a healing material in the extraction sockets. The null hypothesis tested was that PRF would effect the postoperative swelling and pain positively.

## Method

This study was carried in two different Oral and Maxillofacial Surgery Departments. Institutional ethics committee’s approval was obtained for the protocol of the study. [Kırıkkale University, Faculty of dentistry local ethic committee 09/03 (15.04.2013)]. Based on previously treated trial cases we conducted a power analysis (Power and Precision software, Biostat, Englewood, NJ, USA). The findings indicated a minimum sample size of *n* = 50, based on an α of 5 % and a power of 80 %. Considering a possible loss of about 10 % of patients, we used 56 participants.

After preoperative evaluation and obtaining written informed consent, total of 56 patients (23 male, 33 female) ranging from 18-28 years who could follow postoperative instructions were selected for the study.

**Fig. 1:**
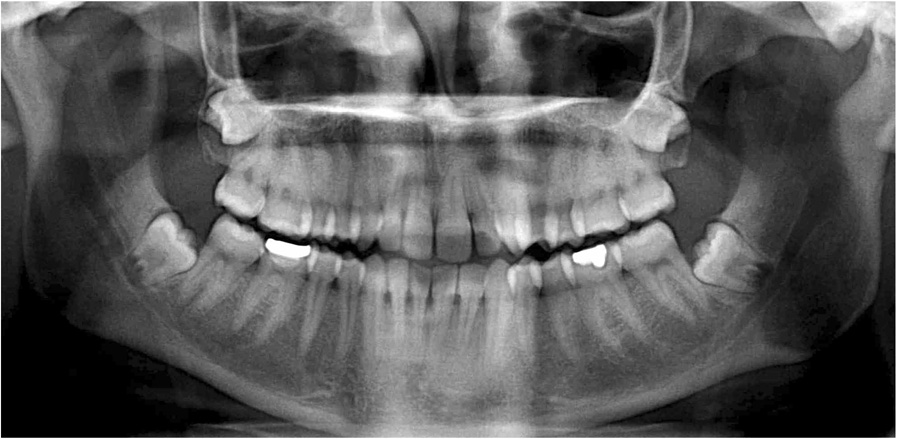
PRF was prepared to place into extraction socket

Clinical inclusion criteria were as follows:

1. Bilateral fully impacted third molars which have the same degree of surgical difficulty comparing one side with the other (Fig. 1).
2. No preexisting medical conditions or no use of medication that would influence or alter wound healing
3. No active pathology associated with the third molars
4. No temporomandibular joint disorder history that would affect the pain sensation after surgery.

Fifty-six patients (23 male-, 33 female) who met the inclusion criteria were selected to participate in this study. Pell and Gregory [18] classification was used to determine the difficulties of the patients included in the study. Of these 56 patients distribution of the classification was as: 20 horizontal, 15 mezioangular, 21 vertical. PRF and the technique were explained to patient and informed consent was taken from all patients.

Patients underwent surgical treatment in accordance with the rules of antisepsis and asepsis. All bilateral third molars surgeries were performed by a single experienced operator in each centers. Prior to the extractions, 10 ml of venous blood was collected from each patient by a surgical nurse and was placed in glass-coated plastic tubes. Tubes were transferred to a centrifuge device and centrifuged for 10 min at 3000 rpm according to Choukroun et al [14].

**Fig. 2:**
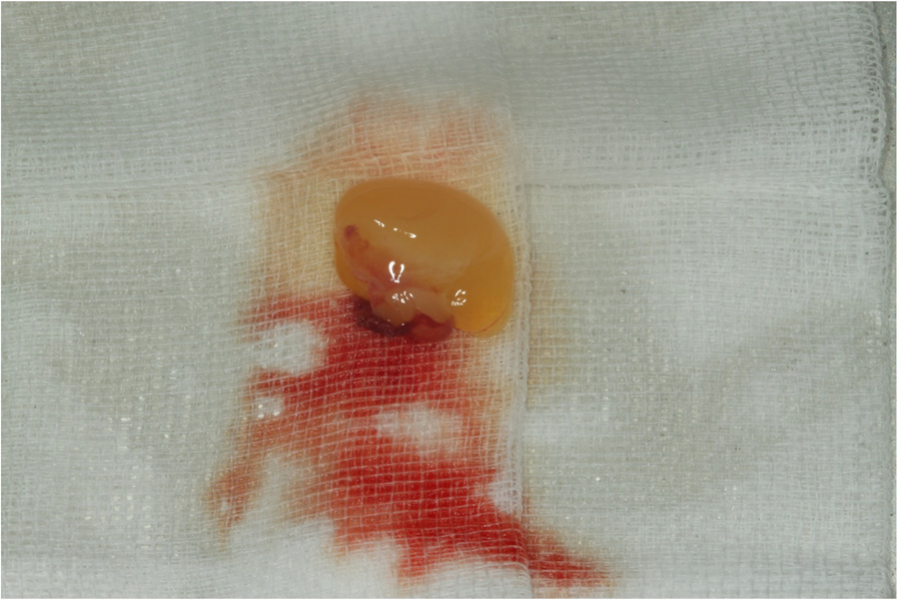
Bilateral impacted third molars which have the same degree of surgical difficulty comparing one side with the other

Following centrifugation, PRF was dissected approximately 2 mm below its connection to the red corpuscle beneath to include remaining platelets, which have been proposed to localize below the junction between PRF and the red corpuscle. Then PRF was squeezed between gauzes to transform into a membrane (Fig. 2).

**Fig. 3:**
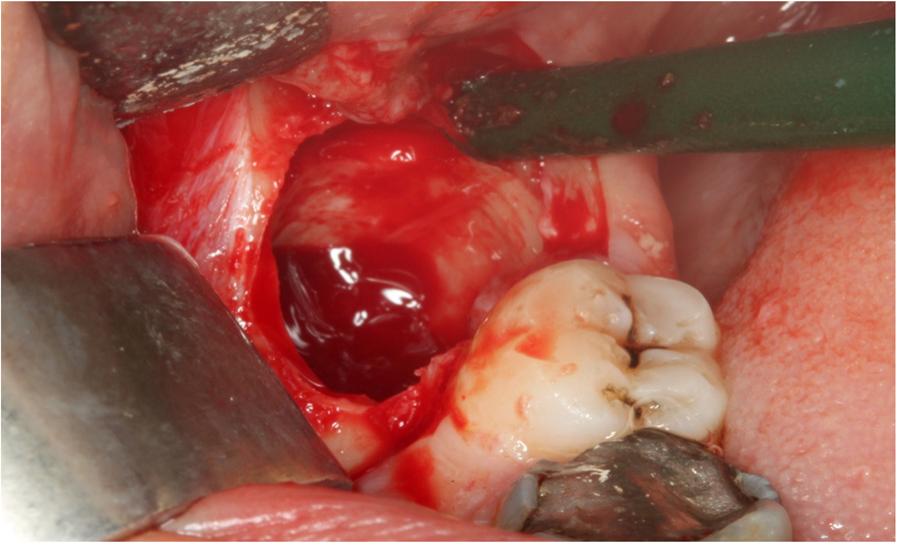
Extraction socket

**Fig. 4:**
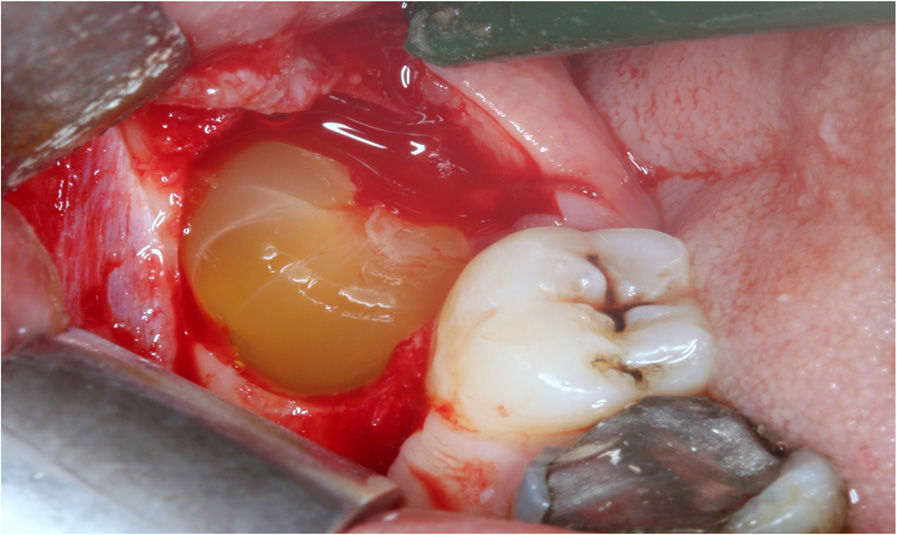
PRF placed into extraction socket on one side

All patients underwent bilateral removal of 3rd molar in single appointment that were the same degree of surgical difficulty. Mandibular and buccal blocks were administered using articaine containing 1:200,000 epinephrine (Ultracain DS; Aventis, Istanbul, Turkey). Horizontal and vertical incisions were performed and a full-thickness mucoperiosteal flap was raised and the tooth was removed with elevators. Following the extraction, the socket was thoroughly irrigated so that pathologic tissue (eg, granulation tissue), follicular remnants, and bony spicules were removed from the cavity (Fig. 3). Following that PRF was placed in the socket on one side which was chosen randomly by coin toss and other side was taken as control group (Fig. 4). Both extraction cavities were primarily closed with 3-4 interrupted sutures using 3.0 silk sutures. Postoperatively, amoxicillin 1000 mg (Alfoxil 1gr; Fako, Istanbul, Turkey) twice daily, paracetamol 500 mg (Vermidon 500 mg; Sandoz, Istanbul, Turkey) thrice daily and chlorhexidine mouthwash thrice daily were administered to all patients for a week.

Patients were blind to the knowledge of PRF placed side in order not to affect visual analog scale (VAS) scores. The pain was evaluated in the postoperative period using a VAS of 100 mm. After the surgery, the patients were instructed to note their postoperative discomfort and/or pain on the scale, which was designed as 0 indicating no pain whereas 100 indicating the worst ever experienced . The evaluation of the postoperative pain was carried out at 24 h, 72 h and seven days after the procedure.

The evaluation of the facial swelling was done by using a horizontal and vertical guide with a flexible ruler. For horizontal guide two points marked at the ear tragus and buccal comissura, and the distance between them was measured and recorded. The same procedure was performed for vertical guide at lateral chantus of the eye and gonion. The evaluation of the postoperative facial swelling was carried out at 24 th hours, 72th hours and seventh day after the procedure. The postoperative evaluations were performed by surgeons that were blinded to the operative procedures, in order to eliminate unwanted bias. The evolution of swelling was evaluated by subtracting the value obtained at each postoperative period by the obtained at baseline [19].

Statistical analysis was performed using the software program SPSS 20.0 (SPSS 20.0 for Windows; SPSS Inc., Chicago, IL, USA), at a significance level of α = 0.05. For analysis of swelling the Shapiro–Wilk test was used to evaluate the distribution of the data (normal or non-normal), the Wilcoxon test for paired samples was used since the data were not normally distributed. Regarding the pain statistical analysis were performed by using one–way analysis of variance (ANOVA). If there were difference between the measurements, the data were analyzed by the Paired Sample test.

## Results

Statistically significant differences were observed in first and third days horizontal measurements between PRF and control side (*p* < 0.05). And more swelling was seen at the control side. The pain scores were measured by using VAS. There were no statistically significant differences observed between groups (Tables 1, 2 and 3).

**Table 1:** Results of Postoperative Swelling (horizontal measurements)

*Horizontal swelling*			
Postoperative evaluation times	Control side Mean(SD)	PRF side Mean (SD)	*P*- value
1^st^ day	4.64 (4.27)	3.28 (3.02)	0.041^*^
3^rd^ day	3.62(3.51)	1.83(2.52)	0.001^*^
7^th^ day	0.73 (1.89)	0.57 (1.87)	0.634

*statistically significant differences amongs groups

**Table 2:** Results of Postoperative Swelling (vertical measurements)

*Vertical swelling*			
Postoperative evaluation times	Control side Mean(SD)	PRF side Mean (SD)	*P*- value
1^st^ day	5.92 (7.42)	5.19 (8.12)	0.306
3^rd^ day	4.00 (6.42)	3.42 (6.55)	0.589
7^th^ day	1.28 (3.95)	0.82 (3.81)	0.061

**Table 3:** Results of Postoperative pain

*VAS scores*			
Postoperative evaluation times	Control side Mean(SD)	PRF side Mean (SD)	*P*- value
1^st^ day	42.84 (29.77)	47.16 (30.59)	0.413
3^rd^ day	26.48 (30.36)	25.50 (29.95)	0.296
7^th^ day	9.41 (16.57)	10.21 (19.75)	0.503

## Discussion

The surgical removal of impacted third molars cause trauma of the soft tissue and bony structures in the oral cavity. The postoperative signs and symptoms of pain, edema and limited mouth opening due to muscle spasm might occur [20, 21].

Fibrin glue [also known as fibrin sealant or fibrin adhesive (platelet rich plasma, PRF)] is a protein based product developed for tissue hemostasis and sealing. Platelet–based materials combine plasma proteins and platelets [22].

PRF described by Choukrouns is prepared naturally without addition of thrombin, and it is hypothesized that PRF has a natural fibrin framework and can protect growth factors from proteolysis [13]. PRF releases high quantities of three main growth factors transforming growth factor β-1 (TGF beta-1), platelet-derived growth factor AB (PDGF-AB), vascular endothelial growth factor (VEGF), and an important coagulation matricellular glycoprotein (thrombospondin-1, TSP-1) during 7 days. Apart from these PRF also secrete EGF, FGF, and three important proinflammatory cytokines- IL-1b, IL-6, and TNF-α which obtained with a simple centrifugation procedure, to stimulate several biological functions such as chemotaxis, angiogenesis, proliferation, differentiation, modulation, thereby representing a possible therapeutic device for a more rapid and effective regeneration of hard and soft tissues [14, 16, 17]. Platelets also play a role in host defense mechanisms at the wound site, by delivering signaling peptides which attract macrophage cells. Platelet concentrates may contain small amounts of leukocytes that synthesize interleukins involved in the non-specific immune reaction [22].

Recently the use of PRF has been proposed as an aid for enhancing regeneration of osseous and epithelial tissues in oral surgery. Several in vitro studies, animal experiments and clinical trials suggested that platelet concentrates may effectively trigger stimulation of osseous and soft tissue regeneration, and reduce inflammation, pain and side effects. The clinical efficacy of PRF in oral surgical procedures is debated as contrasting results have been reported in different clinical procedures [22–25].

Use of PRF in oral cavity has been implicated in different procedures such as extraction socket preservation, intrabony defects, sinus augmentation, and sinus lift procedures for implant placement, bone augmentation, root coverage procedures, and healing in donor site with successful results [22].

Recently some studies evaluated the effect of platelet rich plasma (PRP) to the extraction sockets healing and postoperative complications [26, 27]. Several studies showing animal experiments and clinical trials showed that PRF might affect the regeneration of soft and hard tissue, healing and reduce the side effects [22, 24].

Zhang et al [25] evaluate the influence of PRF on bone regeneration in sinus augmentation. After a healing period of 6 months no statistical differences found between PRF and the control groups.

According to a study by Choukroun et al. a cystic cavity filled with PRF would be totally healed in 2 months instead of the 6 to 12 months required for physiologic healing [22].

There are some controversies in the literature. A study evaluating the effectiveness of PRF in the treatment of intrabony defects of chronic periodontitis patients, thirty-two defects were treated with PRF or a conventional open flap debridement alone. They showed that PRF can be used in the treatment of intrabony defects of chronic periodontitis patients [28]. Lee et al [24] used the PRF for restoration of peri-implant defects in rabbit. Their results showed the possibility of PRF use in bone regeneration.

Contradictory, Aroca et al [15] evaluated that the claimed benefits for soft tissue wound healing induced by PRF membranes and they reported that their results failed to show any beneficial effect of PRF membrane in terms of root coverage or short term wound healing for the treatment of multiple gingival recessions.

Mozatti et al [26] evaluated that the effects of PRP on inflammation process, wound healing, pain and swelling after third molar extraction. They reported that PRP was more effective on wound healing in the extraction socket. Our results supported that PRF was more effective on the swelling in the third day after third molar surgery.

Alissa et al [27] evaluated the influence of PRP on healing of extraction sockets. They reported that PRP may have some benefits in reducing complications such as alveolar osteitis, swelling, pain and improving healing of soft tissue.

Kumar et al [17] investigated the effect of platelet-rich fibrin (PRF) on postoperative pain, swelling, trismus, periodontal healing they concluded that case group had less pain, swelling, and trismus on the first postoperative day compared with the control group. Their results also showed increased and faster periodontal healing in the case group.

In another study by Singh et al [16] they concluded that use of PRF after bilaterally third molar surgeries resulted in less pain compared to control side.

In the present study, PRF was used after third molar extraction, swelling and pain were evaluated. Statistically significant difference was found concerning first and third day horizontal measurements of PRF and control sides with more swelling at the control side (*p* < 0.05). These results are in accordance with Kumar et al [17].

The present study found no significant difference between PRF and control sides in terms of pain which is similar to Singh et al [16], but different from Kumar et al [17] study where the control and PRF groups consisted of different patients. As the results of the study the null hypothesis was partially rejected since there was no positive effect of PRF seen on the pain.

There are some limits to our study; the present study was conducted on bilaterally removed third molars at the same session the results of pain might have been influenced by the control side. Also the use of 3-D optical scanner for the measurements of facial swelling might have given more precise recordings however the funding of the study did not support such expenses. We recommend that further studies investigating this manner should consider the mentioned limitations of the present study.

## Conclusion

As a conclusion, PRF seems to be efficient on postoperative horizontal swelling after third molar surgery. PRF could be used for control swelling after third molar extraction surgery. Studies with a larger sample that will need a bilateral third molar removal that will be extracted in different sessions with a longer follow-up is warranted to obtain a more statistically meaningful results with respect to bone regeneration.

## Abbreviations

NSAID: Non-steroid anti-inflammatory drugs
PRF: Platelet-rich fibrin
PRP: Platelet-rich plasma
VAS: Visual analog scale
